# POLICY SERIES: DOCTORAL PROGRAMS IN GERONTOLOGY: PROVIDING LEADERSHIP IN POLICY AND AGING

**DOI:** 10.1093/geroni/igad104.1772

**Published:** 2023-12-21

**Authors:** Edward Miller, Brian Kaskie

## Abstract

Advancements in education, medicine, wealth, technology, and population health have increased individual life expectancies. Healthy aging is not just a function of healthy genes, however; aging is modified by the broader context, which, in turn, creates opportunities and challenges that must be addressed by society. Lack of organized responses to population aging exacerbate social inequities based on workplace discrimination, displacement, and impoverishment, and contribute to public health inequalities pertaining to disease and disability, isolation, and vulnerability among older adults. Public policy offers one of the most effective ways to address inequities and inequalities such as these. This symposium spotlights four doctoral programs that prepare gerontologists who possess subject matter expertise in aging policy, conduct reliable policy surveillance and sourcing, execute empirical evaluations of policy formation and implementation, and offer well-informed contributions to policy makers who are most involved with supporting and protecting older adults. These well-established academic programs at the University of Massachusetts Boston (Edward Alan Miller, PhD), University of South Florida (Lyndsay Peterson, PhD), University of Southern California (Kathleen Wilbur, PhD), and Miami University (Jennifer M. Kinney, PhD) offer classroom education, research, and service-learning opportunities for students to acquire expertise in aging and public policy. Together, these programs provide models for training a generation of leaders in gerontology who can contribute productively to policy development and debates, illuminate structures and processes of policy making and implementation, and deploy analytic skills needed to evaluate policies relative to multiple and often competing interests.

